# Temporal Data Correlation Providing Enhanced Dynamic Crypto-Ransomware Pre-Encryption Boundary Delineation

**DOI:** 10.3390/s23094355

**Published:** 2023-04-28

**Authors:** Abdullah Alqahtani, Frederick T. Sheldon

**Affiliations:** 1 College of Computer Science and Information Systems, Najran University, Najran 61441, Saudi Arabia; 2 Department of Computer Science, University of Idaho, Moscow, ID 83844, USA; sheldon@uidaho.edu

**Keywords:** crypto-ransomware, data-centric, process-centric, event-based detection, early detection, I/O Request Packet (IRP), malware, Application Programming Interface (API)

## Abstract

Ransomware is a type of malware that employs encryption to target user files, rendering them inaccessible without a decryption key. To combat ransomware, researchers have developed early detection models that seek to identify threats before encryption takes place, often by monitoring the initial calls to cryptographic APIs. However, because encryption is a standard computational activity involved in processes, such as packing, unpacking, and polymorphism, the presence of cryptographic APIs does not necessarily indicate an imminent ransomware attack. Hence, relying solely on cryptographic APIs is insufficient for accurately determining a ransomware pre-encryption boundary. To this end, this paper is devoted to addressing this issue by proposing a Temporal Data Correlation method that associates cryptographic APIs with the I/O Request Packets (IRPs) based on the timestamp for pre-encryption boundary delineation. The process extracts the various features from the pre-encryption dataset for use in early detection model training. Several machine and deep learning classifiers are used to evaluate the accuracy of the proposed solution. Preliminary results show that this newly proposed approach can achieve higher detection accuracy compared to those reported elsewhere.

## 1. Introduction

Ransomware attacks, involving the encryption of victims’ files and subsequent demands for payment in exchange for decryption keys, have emerged as a major concern for organizations. This is due to their escalating sophistication and destructive potential. [1]. The common types or ransomware are crypto-ransomware and locker-ransomware each posing unique threats and necessitating different detection, prevention, and response strategies [1,2,3]. Driven by their profitability and cybercriminals’ growing expertise, ransomware attacks have become more widespread and sophisticated, targeting a diverse range of organizations [1,2]. As a result, organizations must remain vigilant and adopt proactive measures to protect themselves from these devastating attacks.

Ransomware uses the operating system’s own cryptographic libraries to encrypt the files on a victim’s device [1]. It can target several environments and platforms including cloud-based systems, Internet of Things, wireless sensor networks, power grid SCADA (Supervisory Control and Data Acquisition (SCADA)), and intelligent transportation systems [2,3,4,5,6]. Although the nature of the ransomware infection process is similar to other malware categories, the employment of cryptographic means makes the effect of an attack irreversible if the decryption key is not available [7]. Therefore, detection solutions for malware are not necessarily suitable for ransomware because the nature of the target as well as the attack behavior is divergent from that of most other forms of attack [8]. As ransomware attacks target user-related files instead of system-critical resources, existing malware solutions might not be able to detect ransomware due to the different processes and focus [9]. Moreover, as stated above, after the ransomware encryption phase, the attack displays a message to the user asking for a ransom amount promising this is the only way to resolve the unavailability of computational resources and services [1]. Consequently, late detection is not useful after the encryption has completed. Consequently, early detection, also called pre-encryption detection, before the data and executables are encrypted is the lone effective means to protect systems and data against ransomware attacks.

### 1.1. Pre-Encryption Detection

**Figure 1:**
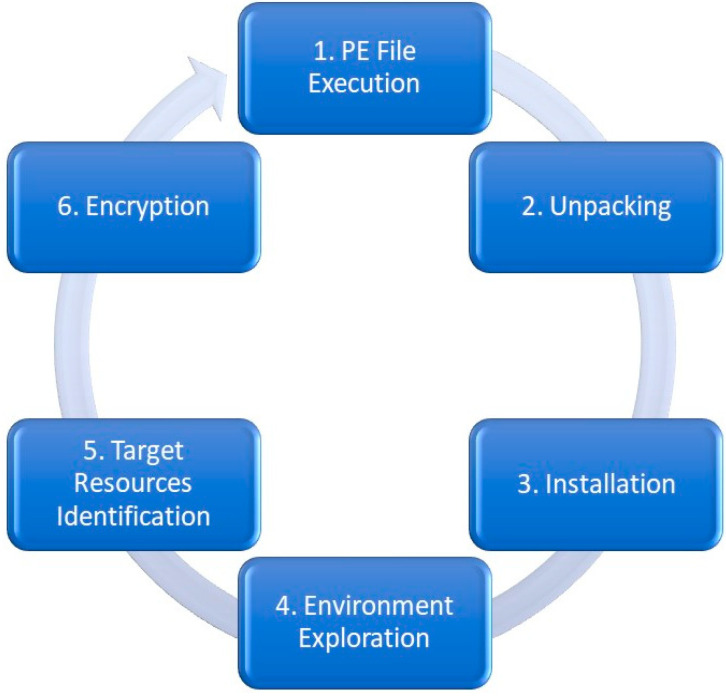
Ransomware’s characteristic pre-encryption lifecycle defining the sequencing of RAW data dependency patterns.

Early detection of ransomware attacks, also called pre-encryption detection, is crucial in minimizing the damage caused by these attacks and reducing the likelihood of successful ransomware infections. By detecting an attack in its early stages, organizations can take steps to isolate the affected systems, prevent the spread of an attack to unaffected resources, and minimize the impact on critical data and systems. Figure 1 shows the stages that ransomware goes through during the pre-encryption phase of its characteristic lifecycle, starting with the Portable Executable (PE) file execution on the victim’s device. The ransomware is unpacked, and the source code is extracted. During this unpacking step, the ransomware could use one or more cryptography-related APIs to decrypt the encrypted payload. This could also involve opening a backdoor comms channel with the Command and Control (C&C) server [10].

Subsequently, the ransomware begins the installation of the malware by exploring the environment on the victim’s device(s). Targeted resources are identified. Finally, the ransomware can *then* encrypt the identified resource files and data. The assumption that the first call of any cryptographic API is highly unlikely. Accordingly, *existing solutions are unable to delineate the boundary of the pre-encryption phase accurately*. This negatively affects the quality of *ransomware activity warning (RAW) data* as it does not capture sufficient ransomware pre-encryption attack patterns, which are necessary for early (pre-encryption) detection.

The first step for early detection (pre-encryption detection) is to identify the pre-encryption phase of a ransomware attack Existing studies pertaining to ransomware pre-encryption detection follow two approaches to determine (delineate) the boundary of the pre-encryption phase, i.e., static and dynamic [11].

#### 1.1.1. Static Pre-Encryption Boundary Delineation

The static pre-encryption studies set a predefined boundary for the pre-encryption phase of the ransomware lifecycle based on a fixed time threshold [12,13]. However, the static approach for defining the pre-encryption boundary is not suitable for detecting sophisticated, evasive ransomware that uses obfuscation and polymorphic techniques to deceive detection. Such obfuscation invalidates the fixed time-based threshold due to encryption start time variability (i.e., ransomware encryption start times can significantly differ, some start earlier, while others start later [11]). There are two implications for using the fixed-time thresholding approach. First, static thresholding may miss the beginning of encryption if the ransomware starts later, and, consequently the pre-encryption data will not be fully captured. This leads to data insufficiency, which negatively impacts the training of early detection models. Second, the static thresholding could exceed the pre-encryption boundary and capture data irrelevant to the pre-encryption data [14]. The inclusion of irrelevant data negatively affects the ability of the detection model to discriminate attack behaviors at the early stages of the ransomware lifecycle [15].

#### 1.1.2. Dynamic Pre-Encryption Boundary Delineation

Dynamic thresholding is another approach used for delineating the pre-encryption boundary of the ransomware attack lifecycle [11,14,16,17]. Unlike the static approach, the dynamic pre-encryption definition does not rely on fixed predefined thresholds for all ransomware samples. Instead, the dynamic approach tracks the boundary of ransomware pre-encryption during the runtime based on the calling of the cryptographic APIs [17]. The existing solutions that follow this approach build pre-encryption boundary vectors that contain the cryptographic-related APIs. This vector is used as a reference from which upcoming API call events are compared. When a match occurs, the end of the pre-encryption phase is marked accordingly.

However, calling a cryptographic API does not necessarily indicate the beginning of a malicious encryption cycle [17]. For example, benign applications commonly call cryptographic APIs for purposes other than encryption involving packing, unpacking, and obfuscation, which are typically seen from malicious activities. Such nonencryption activities always occur at the very beginning of the ransomware installation. This is a warning signal that occurs way before the malicious use of encryption takes place. Consequently, relying solely on crypto-APIs to define the boundary of the pre-encryption phase results in an early, premature cutoff of data collection. This limitation deprives the detection model of essential early attack patterns necessary for accurate ransomware detection. As a result, the data used for training the model become inadequate, leading to a decline in detection accuracy.

### 1.2. Accurately Defining Pre-Encryption Boundaries

As discussed above, the inability to capture the proper amount of pre-encryption data leads to insufficient training data. To address such an issue, this paper characterizes the utilization of the temporal correlation between the API data and the I/O Request Packet (IRP) data for accurate pre-encryption boundary delineation. Temporal correlation between APIs and I/O Request Packets (IRPs) in ransomware analysis refers to the degree of association between the usage of specific APIs and the appearance of corresponding IRPs over time [17,18]. A high temporal correlation between APIs and IRPs would imply that when a particular API is used, the corresponding IRP tends to appear with a consistent time lag or lead. This can help analysts identify patterns in ransomware behavior and potentially develop more effective detection and mitigation strategies. By studying the temporal correlation between APIs and IRPs, we can gain insights into the relationships between ransomware’s technical execution and its observable effects. This understanding can improve the ability to delineate the pre-encryption phase of ransomware more effectively.

Ransomware that encrypts user data utilizes the cryptography-related APIs of the underlying operating system [19]. Naturally, the ransomware would access the system files where the targeted files are located summoning IRP functions that can be captured by monitoring the invoking process(es). As the calls of the IRP and API happen nearly at the same time, the temporal correlation that relates the IRP and API data is determined based on their timestamps. By accurately defining the pre-encryption boundary for the ransomware lifecycle, more data can be captured, and sufficiently early attack patterns can be recorded, which will certainly improve early detection accuracy.

### 1.3. Research Contribution

Herein, the temporal correlation was integrated into the feature extraction stage of ransomware early detection modeling. To this end, the contribution of this paper is three-fold. We

- propose a Temporal Data Correlation (TDC) method that associates the cryptographic APIs with the IRPs based on the timestamp for pre-encryption boundary delineation,
- incorporate the TDC method into the Improved Pre-encryption Feature Extraction (IPFE) technique, which focuses on pre-encryption features,
- evaluate the accuracy of this early detection model trained using the pre-encryption data as well as using the common performance evaluation metrics.

Note that in this paper, crypto-ransomware and ransomware are used interchangeably, unless stated otherwise. The rest of this paper is organized as follows. Section 2 details the design of the methods and techniques proposed. Section 3 explains and discusses the results obtained by the proposed technique as well as a comparison with related works. The paper closes with a summary and conclusion.

### 1.4. Related Works

As pointed out above, ransomware can be classified into two types: locking-ransomware and crypto-ransomware [8]. Unlike locking-ransomware, which can be easily bypassed, the effects of crypto-ransomware are permanent because it uses encryption on user files. Without the decryption key, it becomes difficult or even impossible for the victim to regain access to their data [12]. Therefore, it is essential to detect this type of malware before it begins encrypting user files and data.

Various studies have examined the irreversible impact of ransomware attacks and suggested methods for identifying them. These methods can be classified into two groups: data-centric and process-centric. Data-centric ransomware detection keeps track of digital assets on the victim’s computer and triggers an alert when an unusual change is observed [8]. Techniques, such as decoy, entropy, and similarity, are used by data-centric solutions to monitor file structure before and after access [20,21,22,23].

However, this approach cannot distinguish between changes made by ransomware and those made by harmless programs, leading to a high number of false alarms [24,25,26]. More significantly, this method does not provide complete protection against ransomware attacks because it sacrifices some files that may be more valuable than the remaining data [8,25]. As a result, the data-centric approach is not effective for the early detection of ransomware.

In contrast, process-centric research monitors the behavior of active processes to gather various types of behavioral data. These data are then used to train different machine learning classifiers, such as Random Forests and Naïve Bayes [27,28,29]. However, these solutions rely on complete runtime data, including pre-encryption and post-encryption data, to train the detection model [30,31]. This approach assumes that complete attack patterns are available at the time of detection, which is not true for the early detection of crypto-ransomware when attack data are not fully available [3].

Another type of process-centric ransomware detection involves monitoring various resources on the local machine, such as CPU, network, I/O buffer, and memory [20,32,33,34]. When encryption-related events are detected, an alarm is triggered by the detection system. However, relying on ad hoc events to detect crypto-ransomware attacks increases the rate of false alarms because these events are not exclusive to crypto-ransomware. Some normal programs also trigger similar events [14]. Furthermore, these ad hoc events may be triggered after encryption has occurred making this approach ineffective for early detection [14]. Therefore, effective detection must take place during the early stages (pre-encryption) before ransomware begins its primary sabotage, i.e., encryption.

Identifying the pre-encryption boundary is crucial for effective early detection solutions. Several studies [8,12,13,30] have been conducted to investigate how such a boundary could be determined. They rely on fixed thresholds as a cut-off to separate the pre-encryption phase from the later phases. These thresholds fall under two categories: time-based (temporal) and API-based. Time-based thresholds [13,30] define a fixed time for each ransomware to run and collect data before execution is interrupted. API-based thresholds [8,30] rely on a set of pre-defined cryptography-related APIs and use them to label the pre-encryption boundary.

Based on the literature discussed above, the existing works lack an effective mechanism for the early (pre-encryption) detection of ransomware. They were built based on an assumption that the pre-encryption phase of the attack is fixed and can be defined based on static thresholds, such as time or APIs. However, fixed thresholding, whether time-based or API-based, may not accurately detect the start of encryption in ransomware attacks. This may result in an incorrect representation of the pre-encryption phase and hinder the ability of detection solutions to identify attacks before encryption takes place. Fixed time-based thresholding assumes that all instances begin encryption before a specified time. However, this assumption is often incorrect due to obfuscation techniques that create varying attack behaviors. Similarly, relying solely on the first cryptography API call to determine pre-encryption boundaries can be misleading. For example, the first cryptography API call may not be related to the user’s file encryption and could instead be used for standard tasks, such as unpacking or decrypting metamorphic payloads, before malicious activities begin.

Our study aims to address the limitations of fixed thresholding in detecting the start of encryption in crypto-ransomware attacks by investigating a more accurate method for delineating pre-encryption boundaries. Unlike existing approaches, our solution does not rely on fixed thresholds. Instead, it examines the correlation between cryptography APIs and IRPs based on timestamps. This correlation allows for a more precise identification of the starting point of actual file encryption. We expect that our model can more accurately determine when actual ransomware encryption begins, regardless of the various APIs or time constraints. This starting point serves as the boundary separating the pre-encryption phase from subsequent phases of ransomware attacks.

## 2. Methodology

The methodology adopted here as well as the design of the proposed techniques is detailed. The proposed model consists of three main components: (i) Temporal Data Correlation, (ii) Pre-encryption Feature Extraction, and (iii) Training and Testing of our early detection model. These components are designed so that the output of the preceding step becomes the input to the succeeding step. The following subsections elaborate the data and design of those components.

### 2.1. Dataset

The ransomware Portable Excitable (PE) files can be downloaded from the Virusshare (https://www.virusshare.com) site, a public repository used by related studies including [8,12,27,30,35,36,37]. The corpus consists of 39,378 ransomware samples representing different families, such as CryptoWall, Petya, and WannaCry. Moreover, 9732 benign applications were downloaded from https://www.informer.com, a popular application repository used by many ransomware and malware studies including [1,12,27,38,39,40]. The files were executed in the Cuckoo Sandbox (2.0.7) virtual platform one by one [12,30,41,42,43]. After running each sample in a guest virtual machine, which is used to represent a victim, the sandbox hooks the process created by the program under observation and captures the APIs and IRPs into a trace file.

**Figure 2:**
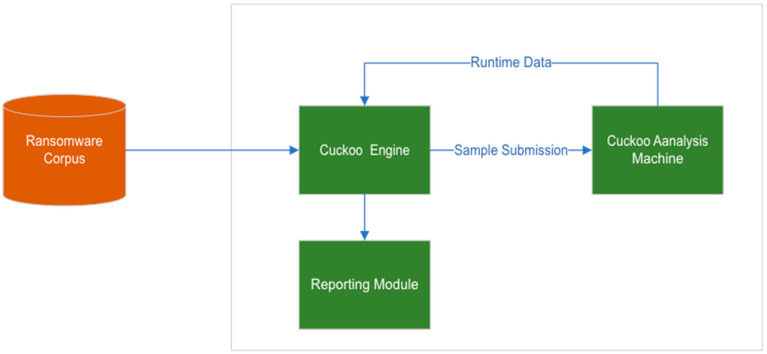
Cuckoo Sandbox Analysis Environment.

To collect attack patterns from the runtime data of samples in the dataset, each sample was analyzed dynamically on an analysis platform called *Cuckoo Sandbox (2.0.7)*. Figure 2 shows a diagram of the Cuckoo Sandbox analysis environment. Cuckoo Sandbox is an open-source framework for dynamically analyzing malware in a controlled and isolated environment. It creates virtual machines to emulate different systems and executes malware samples inside them to analyze their behavior. Cuckoo Sandbox can analyze various types of malicious files and websites and trace API calls and general behavior, including analyzing network traffic. The Sandbox is highly customizable and can integrate additional tools for enhanced analysis capabilities, such as advanced memory analysis through Volatility or YARA. Cuckoo also has embedded scripts that simulate basic user activities.

Like the related works [8,13,30] that adopted the same procedure as suggested by [44], we used Cuckoo Sandbox to run the ransomware samples and collect the runtime data. When a ransomware sample is submitted, the sandbox sends it to the guest machine, which imitates the victim’s device. Then, the Sandbox’s agent within the guest machine executes the submitted sample. When the sample executes, a process in the guest machine is created for it. Then, the sandbox uses a hooking utility to intercept the ransomware running process in the guest machine and captures its runtime data including API calls, which were stored in a trace file designated for that sample. The following steps were used when conducting the dynamic analysis:

1. A command line utility called ‘submit’ within the sandbox was used to submit the malware sample to Cuckoo Sandbox.
2. Then, the Cuckoo Sandbox automatically began the dynamic analysis process. Cuckoo created a new virtual machine (VM) instance from the clean snapshot, executed the malware sample within the isolated environment, and monitored the malwares’ behavior.
3. The analysis progress could be monitored in real time through the Cuckoo web interface or command line output.
4. Cuckoo logged the actions performed by the malware, such as file system changes, network activity, and API calls into a JSON file.
5. Once the analysis was complete, Cuckoo generated a detailed report containing information about the malware’s behavior and impact on the system.
6. The guest machine was then restored to its clean state, ready for analysis of the next ransomware sample. This step ensured that the behavior of the next sample was not influenced by any previous infections.

Cuckoo generated comprehensive reports, in a JSON format, following malware analysis, providing insights into the malware’s behavior and potential impact on a system. The reports encompass both static and dynamic analysis information, including file details, metadata, extracted strings, behavioral summaries, API calls, process trees, file system and registry activities, network interactions, and memory analysis. Additionally, the reports offer screenshots, dropped files, and extracted Indicators of Compromise (IoCs), facilitating a deeper understanding of the malware’s functionality, characteristics, and potential mitigation strategies. These JSON files comprise the corpus from which the dataset was built, and features were extracted and selected before being used to train the detection model. Figure 2 shows the architecture of the crypto-ransomware dynamic analysis. After each run, the guest machine was reset to its original uninfected state to ensure that subsequent samples would not be influenced by previous infections. From each trace file’s runtime data, only API calls and their parameters were kept, while all other data were discarded.

### 2.2. Temporal Data Correlation Method

Based on the dataset collected from executing the Portable Excitable (PE) files in the Sandbox, the Temporal Data Correlation (TDC) method was developed. The method extracted cryptographic APIs and IRPs by looking into the timestamp. The cryptographic APIs and IRPs that shared the same timestamp were paired as a group (vector). This is because multiple APIs and IRPs were called at the same timestamp. At the beginning, the vector containing cryptographic APIs was built according to [8]. Then, this vector was used as input to the TDC, which used it as a filter for selecting the cryptographic APIs and filtering out the irrelevant ones.

As pointed out above, the temporal correlation was performed based on the timestamp attached with both APIs and IRPs. As multiple APIs/IRPs could share the same timestamp, the data were grouped based on the timestamp. After that, API arguments (input and output parameters) were used to check whether a particular cryptographic API interacted with the file system. This was performed by looking into the presence or absence of file handlers. If the API used the file handlers, it would be kept in the API/IRP group and removed otherwise. Based on such criteria, the first API/IRP match was considered as the boundary that ends the pre-encryption phase and starts the encryption phase within the ransomware lifecycle. Then, the set of APIs/IRPs that satisfy the criteria were combined into a bidimensional pre-encryption vector, where the pre-encryption boundary for each ransomware was determined.

### 2.3. Improved Pre-Encryption Feature Extraction (IPFE) Technique

After the pre-encryption vector was constructed, it was used to determine the pre-encryption boundary for each trace file in the corpus. The data that came before this boundary were extracted into another trace file called the pre-encryption trace (PT) file such that each program in the original corpus had its own PT file. In the PT files, only API calls were included, while other types of data were removed. These PT files were used as input for the feature extraction stage.

During feature extraction, the annotated Term Frequency Inverse Document Frequency (TF-IDF) technique [8] was used. The TF-IDF gives more weight to features related to the pre-encryption phase of the ransomware lifecycle. The general formula used to compute the Term Frequency-Inverse Document Frequency (TF-IDF) is expressed here [8]:

(1) $$w(api_{k}^{j})=tf(api_{k}^{j})\cdot \log\frac{N}{idf\left(api_{k}\right)}$$

where $api_{k}$ represents the *k*th API and $tf(api_{k}^{j})$ computes a term frequency at the ransomware’s PT file level, $r_{j}$. Moreover, $idf\left(api_{k}\right)$ computes the inverse document frequency on the corpus level to determine the number of ransomware samples, $r_{j}$, that used an $api_{k}$ at least once. N is the total number of ransomware instances in the corpus.

The features extracted by the IPFE technique were then used to train a set of machine learning classifiers for *ransomware early detection (RED)*. The classifiers used in this study were a mix of traditional and deep learning approaches. In particular, the Logistic Regression, Support Vector Machines, Deep Belief Networks, and Convolutional Neural Networks were used for testing the efficacy of the features extracted by IPFE for ransomware early detection.

### 2.4. Experimental Environment

The ransomware analysis was carried out in an isolated environment using the Cuckoo Sandbox installed on a PC with Intel^®^ Core^TM^ i7-4790 CPU @ 3.60 GHZ and 32 GB RAM. The Cuckoo Sandbox platform was developed based on the guidelines described in [29]. Moreover, the Cuckoo Sandbox was installed inside an Oracle Virtual Box. A Linux Ubuntu 4.4.0-59 generic machine was created as a Cuckoo platform inside Virtualbox, where Cuckoo Sandbox software and related packages were installed. In addition, a Windows 7 guest machine was created inside the Cuckoo Sandbox platform. In the virtual machine, a collection of user-related applications and files were established to give the appearance of a genuine computer. The virtual machine had several applications installed, including Microsoft Office, Adobe Acrobat Reader, Google Chrome, and Mozilla Firefox. Moreover, various nonsystem folders were established in different parts of the virtual machine’s file system. These folders contained approximately 1647 files, including Microsoft Word documents, Excel sheets, PowerPoint presentations, Visio files, PDFs, JPG images, and short video files.

The ransomware and nonmalicious programs were individually executed in the Sandbox environment, and their data were recorded into a separate trace file for each program. After each execution, the virtual machine was returned to its original, pristine state. The collected data were cleaned to eliminate any irrelevant information and then used as input for the TDC and IPFE feature extraction technique phase. The techniques, along with the results and analysis, were implemented using Python libraries, such as Sklearn, Pan-das, and Numpy.

## 3. Results and Discussion

The performance of our proposed *Improved Pre-encryption Feature Extraction* (IPFE) technique was evaluated using a dataset that was divided into a training set and a testing set using 10-fold cross-validation. The IPFE technique is utilized to extract features from the data, which were then used to train various machine learning classifiers, including Support Vector Machine (SVM), Logistic Regression (LR), Deep Belief Networks (DBN), Convolutional Neural Network (CNN), and Multi-layer Perceptron (MLP). The test set was employed to assess the classification performance of each classifier based on the extracted features using metrics, such as accuracy (ACC), F score (F1), precision, recall, and the Area under the ROC Curve (ROC-AUC). Equations (2)–(5) outline the calculation of these metrics.

(2) $$\begin{array}{ll} \mathrm{Accuracy}\;(\mathrm{ACC}) & ACC=\frac{TP+TN}{TP+TN+FP+FN} \end{array}$$

(3) $$\begin{array}{ll} F1 & F1=\frac{2TP+}{2TP+FP+FN}\; \end{array}$$

(4) $$\begin{array}{ll} \mathrm{Precision} & Precision=\frac{TP}{TP+FP} \end{array}$$

(5) $$\begin{array}{ll} \mathrm{Recall} & Recall=\frac{TP}{FP+FN}\; \end{array}$$

where FP, TP, FN, and TN denote false-positive, true-positive, false-negative, and true-negative, respectively.

The experimental results of the classifier trained using the extracted features using our IPFE technique are represented in Table 1. We used various evaluation metrics including Recall, Precision, F score (F1), accuracy (ACC), and False-Positive Rate (FPR). The recall results depict that the MLP classifier achieved 0.947, which is the lowest value among the results, while the SVM classifier achieved the highest value of recall (99). According to the precision of classifiers, our results show that the IPFE classifier’s precision ranged between 0.9 for MLP and 0.942 for DBN. Similarly, the results of the F1 values range between 0.932 and 0.952 for MLP and DBN, respectively. In the case of classification accuracy, the results of the classifiers range between 0.892 for LR and 0.946 for DBN. The values for the ROC_AUC range between 0.812 and 0.893 for MLP and DBN, respectively.

**Figure 3:**
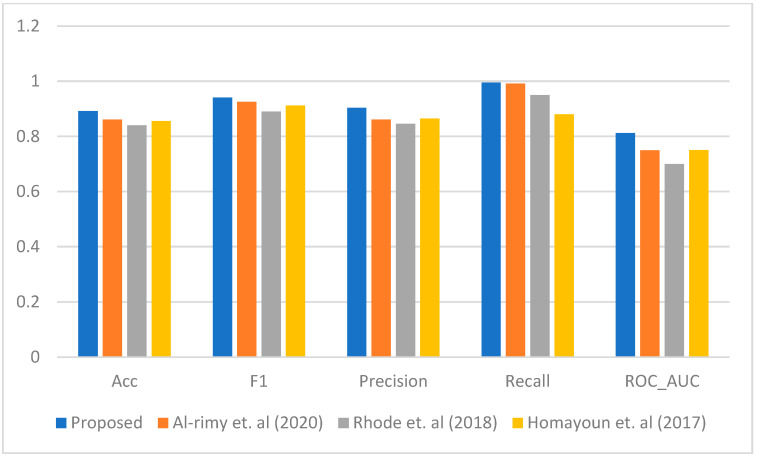
The performance comparison between the proposed IPFE with related techniques (Al-rimy et al. (2020) [8], Rhode et al. (2018) [30], and Homayoun et al. (2017) [13]) using LR.

**Figure 4:**
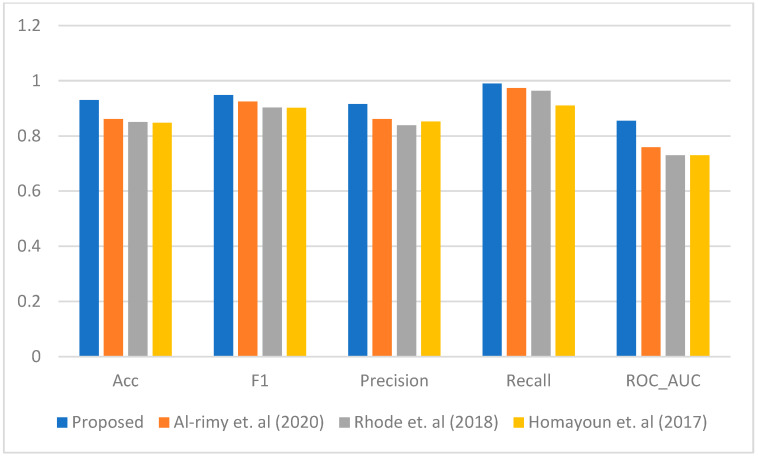
The performance comparison between the proposed IPFE with related techniques (Al-rimy et al. (2020) [8], Rhode et al. (2018) [30], and Homayoun et al. (2017) [13]) SVM.

**Figure 5:**
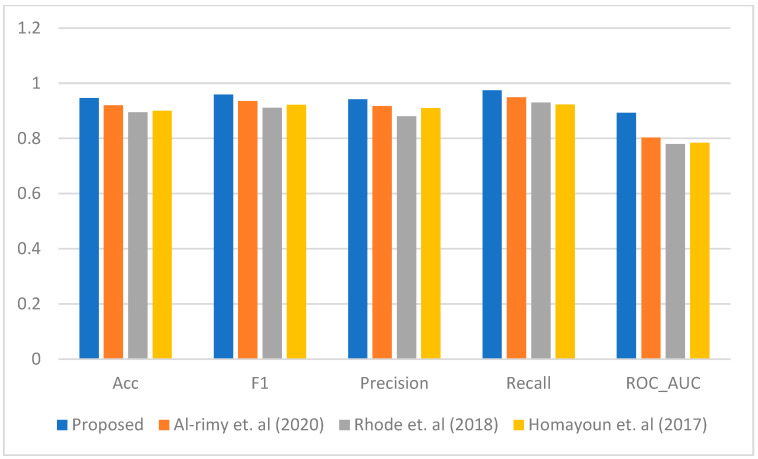
The performance comparison between the proposed IPFE with related techniques (Al-rimy et al. (2020) [8], Rhode et al. (2018) [30], and Homayoun et al. (2017) [13]) using DBN.

**Figure 6:**
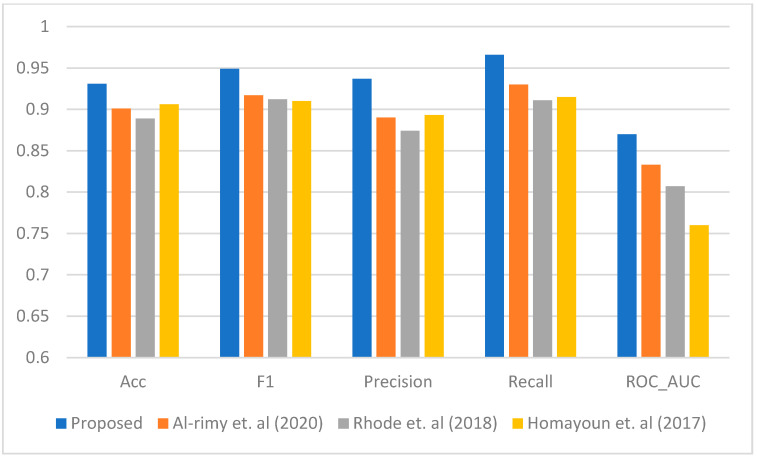
The performance comparison between the proposed IPFE with related techniques (Al-rimy et al. (2020) [8], Rhode et al. (2018) [30], and Homayoun et al. (2017) [13]) using CNN.

**Figure 7:**
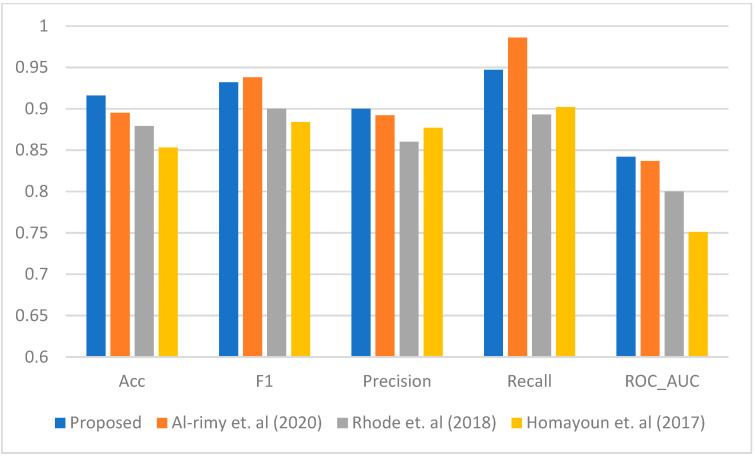
The performance comparison between the proposed IPFE with related techniques (Al-rimy et al. (2020) [8], Rhode et al. (2018) [30], and Homayoun et al. (2017) [13]) using MLP.

Figure 3, Figure 4, Figure 5, Figure 6 and Figure 7 represent the results of the IPFE technique in comparison with the various state-of-the-art models. The purpose of this comparison it to show the improvement achieved by the IPFE technique compared to the related work from the literature. Herein, we chose the studies from [8,13,30] as they follow the same approach and investigate the same problem as we do here. We note that the results of the recall, precision, F1, accuracy, and ROC_AUC classifications from our IPFE technique were improved compared to the conventional techniques that we considered against all other classifiers. Therefore, the temporal correlation among the APIs and IRPs utilized in our IPFE technique can identify a new *key* behavioral aspect of the crypto-ransomware attack lifecycle in an earlier stage better than those other methods, *under the assumption that the downloaded data are representative of comparable testing*. Again, consider Table 1 and Figure 3, Figure 4, Figure 5, Figure 6 and Figure 7 regarding these claims.

This improvement by the IPFE technique to obtain and utilize more data as compared to other models results in a better characterization of discrete stages in the ransomware lifecycle. IPFE tracks the beginning point of the encryption processes for each instance individually. Thus, the IPFE technique takes advantage of the complimentary nature that the various instances display in the dataset, giving it the opportunity to extract more pre-encryption data that result in a better and more accurate characterization. In other words, although some instances start the encryption very early, there are other instances that start the encryption later. Therefore, the dynamic thresholding compensates for the lack of information about the instances that start the encryption early using the information gathered by the instances that start the encryption cycle later.

## 4. Summary, Conclusions, and Future Research

This paper presents a novel approach for early ransomware detection by leveraging temporal correlation between API data and I/O Request Packet (IRP) data for accurate pre-encryption boundary delineation. The proposed Temporal Data Correlation (TDC) method effectively identifies whether a specific cryptographic API is involved in the ransomware encryption process. By constructing a vector of API–IRP pairs, the TDC method represents the pre-encryption phase of the ransomware lifecycle, which is crucial for early detection. The IPFE technique employed in this study aids in extracting the pre-encryption features that are essential for training various machine learning and deep learning classifiers. These classifiers were then utilized in the development of a ransomware early detection model, which aimed to identify ransomware attacks even before the encryption process commences.

The experimental results of our study substantiate the effectiveness of the proposed solution, demonstrating its capability to detect ransomware attacks in their early stages. This innovative approach has the potential not only to enhance the security of computer systems but also to mitigate the detrimental consequences of ransomware attacks on users and organizations. By further refining and developing the proposed techniques, it is anticipated that the early detection of ransomware attacks will become increasingly reliable, contributing to the advancement of cybersecurity ransomware countermeasures and the protection of valuable digital assets.

For future work, this research can be extended in several ways. One possibility is to improve the IPFE technique by incorporating redundancy estimation to extract unique features and reduce data dimensionality. Another potential improvement is to add a relevancy score calculation coefficient to the IPFE technique, which would help determine the significance of features with respect to the target label. By incorporating these mechanisms, the IPFE technique is expected to extract a compact set of relevant and nonredundant features, thereby reducing model complexity and increasing its efficiency. In terms of improving the TDC method, one possibility is to add the capability to analyze anti-analysis ransomware samples. This type of ransomware examines its environment, and if it detects an analysis tool artifact, halts its execution or changes its behavior to conceal its real intent. As a result, the analysis sandbox may not be able to capture the ransomware’s behavior accurately. Addressing these challenges will be a focus of our future research.

## Figures and Tables

**Table 1:** Performance of the IPFE in terms of accuracy (ACC), F1, Precision, Recall, and ROC_AUC.

	LR	SVM	DBN	CNN	MLP
ACC	0.892	0.93	0.946	0.931	0.916
F1	0.941	0.948	0.959	0.949	0.932
Precision	0.904	0.916	0.942	0.937	0.9
Recall	0.995	0.99	0.974	0.966	0.947
ROC_AUC	0.812	0.855	0.893	0.87	0.842

## Data Availability

Data is unavailable due to privacy.
